# Distinct synovial immunopathology in Behçet disease and psoriatic arthritis

**DOI:** 10.1186/ar2608

**Published:** 2009-02-06

**Authors:** Juan D Cañete, Raquel Celis, Troy Noordenbos, Conchita Moll, Jose A Gómez-Puerta, Pilar Pizcueta, Antonio Palacin, Paul P Tak, Raimon Sanmartí, Dominique Baeten

**Affiliations:** 1 Arthritis Unit, Department of Rheumatology, Hospital Clinic de Barcelona and IDIBAPS, Villaroel 170, Barcelona, 08036, Spain; 2 Clinical Immunology and Rheumatology, Academic Medical Center/University of Amsterdam, Meibergdreef 9, Amsterdam, 1105 AZ, The Netherlands; 3 Immunology Unit, Department of Cell Biology and Pathology, Calle Casanova 143, Universidad de Barcelona, 08036, Spain; 4 Department of Pathology, Hospital Clinic de Barcelona, Villaroel 170, Barcelona, 08036, Spain

## Abstract

**Introduction:**

The aim of the study was to investigate synovial immunopathology differences between early Behçet disease (BD) and psoriatic arthritis (PsA).

**Methods:**

Needle arthroscopy of an inflamed knee joint was performed in patients with early untreated BD (n = 8) and PsA (n = 9). Synovial fluid (SF) was collected for cytokines, perforin, and granzyme analysis. Eight synovial biopsies per patient were obtained for immunohistochemical analysis of the cellular infiltrate (T cells, natural killer cells, macrophages, B cells, plasma cells, mast cells, and neutrophils), blood vessels as well as expression of perforin and granzyme. The stained slides were evaluated by digital image analysis.

**Results:**

The global degree of synovial inflammation was similar in the two types of arthritis. In the analysis of the innate immune cell infiltration, there was a striking neutrophilic inflammation in BD synovitis whereas PsA displayed significantly higher numbers of cells positive for c-kit, a marker of mast cells. As for lymphocytes, CD3^+ ^T cells, but neither CD20^+ ^B cells nor CD138^+ ^plasma cells, were significantly increased in BD versus PsA. Further analysis of the T-lymphocyte population showed no clear shift in CD4/CD8 ratio or Th1/Th2/Th17 profile. The SF levels of perforin, an effector molecule of cytotoxic cells, displayed a significant four- to fivefold increase in BD.

**Conclusions:**

This systematic comparative analysis of early untreated synovitis identifies neutrophils and T lymphocytes as important infiltrating cell populations in BD. Increased levels of perforin in BD suggest the relevance of cytotoxicity in this disease.

## Introduction

Behçet disease (BD) is a systemic inflammatory disorder with oral and/or genital ulcerations, uveitis, and skin lesions as prototypic clinical symptoms [1]. The systemic nature of the disease is emphasized by the potential involvement of the central nervous system, the vascular system, the gut, and the kidney. Up to half of the patients with BD also display rheumatic features. The arthritis is usually monoarticular, intermittent, and not deforming and affects mainly knees or ankles. Less common rheumatic features are enthesitis, spondylitis, and sacroiliitis. This pattern of rheumatic inflammation as well as the association with eye, gut, and skin involvement display clinical similarities with spondyloarthritis (SpA) in general and psoriatic arthritis (PsA) in particular. Although the pathogenesis of BD is still poorly understood, the association with class I major histocompatibility complex molecules (HLA-B51 in BD and HLA-B27 in SpA) and the response to tumor necrosis factor (TNF) blockers in both diseases further support the possibility of common pathophysiological pathways.

Previous studies in psoriatic and non-psoriatic SpA and rheumatoid arthritis (RA) have indicated that detailed synovial histopathology can help to reveal differences in cellular and molecular immunopathology which are of interest for differential diagnosis, classification, and pathogenesis of the different types of arthritis [2-4]. Although joint involvement is clinically well recognized, few histologic studies have focused on the synovial features in BD [5-7]. Here, to explore the immunopathology of BD, we performed a detailed comparative study of early untreated synovitis in BD versus PsA.

## Materials and methods

### Patients and samples

Eight patients with early untreated BD and nine patients with early untreated PsA gave written informed consent to participate in the study as approved by the local ethics committee. All untreated patients from our early arthritis clinic undergoing diagnostic or therapeutic knee arthroscopy and fulfilling either the International Study Group for Behçet's disease criteria for BD [8] or the CASPAR (classification of psoriatic arthritis) criteria for PsA [9] were included. All BD patients had recurrent oral and genital aphtosis and folliculitis, five had a positive pathergy test, two had uveitis, and one developed retinal vasculitis. All patients of both study cohorts had early disease (values of median [range] of disease duration since diagnosis was made in the early arthritis clinic were 1.0 [0.2 to 7.9] months in BD and 4.0 [1.7 to 13.5] months in PsA) and were disease-modifying antirheumatic drug (DMARD)-naïve and corticosteroid-naïve. All patients had active joint disease with at least one swollen knee joint (five monoarthritides and three oligoarthritides in BD and two monoarthritides and seven oligoarthritides in PsA). Median erythrocyte sedimentation rate (millimeters per hour) values were 50 (range of 12 to 113) in BD and 32 (10 to 85) in PsA. Median C-reactive protein (milligrams per deciliter) values were 6.8 (2 to 15.5) in BD and 2.3 (1.5 to 9.4) in PsA. Needle arthroscopy of the clinically inflamed knee joint was performed in all patients, with a 2.7-mm arthroscope (Olympus^®^; Barcelona, Spain). Synovial fluid (SF) was collected for cytokine analysis, and eight synovial biopsies per patient were obtained for immunohistochemical analysis.

### Immunohistochemistry

The synovial biopsies were embedded in paraffin, sectioned, and subjected to antigen retrieval by cooking when required. The slides were subsequently stained with an automated immunostainer (TechMate 500 Plus; Dako, Cambridge, UK) using the following monoclonal antibodies: anti-CD3 (clone PS1; Novocastra, Newcastle, UK), anti-CD4 (clone 1F6; Novocastra), anti-CD8 (clone 4B11; Novocastra), anti-CD20 (clone L26; Dako), anti-CD15 (clone BY87; Novocastra), anti-CD31 (clone JC70A; Dako), anti-CD56 (clone 123C3; Monosan, Uden, The Netherlands), anti-CD68 (clone KP-1; Dako), anti-CD117 (mast cells, rabbit anti-human polyclonal antibody; Dako), anti-CD138 (clone B-B4; Santa Cruz Biotechnology, Inc., San Diego, CA, USA), anti-granzyme B (clone GrB7; Monosan), and anti-perforin (clone 5B10; Novocastra). As a negative control, the primary antibodies were substituted by isotype- and concentration-matched control antibodies. The primary antibodies were subsequently detected by an avidin-biotin-peroxidase-based method (Envision System; Dako) and an aminoethylcarbazole color reaction (Sigma-Aldrich, St. Louis, MO, USA) as described previously in detail [2-4,10]. Finally, the slides were counterstained with hematoxylin.

### Digital image analysis

The stained slides were scored by digital image analysis by an independent observer (RC) who was blinded to diagnosis and clinical data. Each stained slide in its entirety was scored by dividing it in different regions. Within each region, the number of stained cells per area as well as the percentage of stained cells were measured in at least 20 high-power fields using the AnalySIS^® ^Imaging processing program (Olympus^®^) as described previously in detail [11]. The presence of grade-3 lymphoid aggregates, as defined previously [12-15], was assessed on consecutive sections stained for CD3 and CD20.

### Synovial fluid analysis

SF was collected at the time of arthroscopy for assessment of total cellular count and number of neutrophils. SF levels of interferon-gamma (IFN-γ), TNF-α, interleukin (IL)-2, IL-4, IL-6, and IL-10 were quantified using a multiplex assay in accordance with the instructions of the manufacturer (Cytometric Bead Assay; BD Biosciences, San Jose, CA, USA). Perforin (Abcam, Cambridge, UK), granzyme B (Abcam), IL-8 (R&D Systems, Abingdon, UK), and IL-17 (R&D Systems) levels in SF were assessed by enzyme-linked immunosorbent assay (ELISA) as indicated by the manufacturer.

### Statistical analysis

Data were analyzed using the SPSS 10.0 statistical program (SPSS Inc., Chicago, IL, USA). As the data are non-parametric, they are represented as median (range). Comparisons were performed with the non-parametric Mann-Whitney *U *test. The level of statistical significance was established at a *P *value of less than 0.05.

## Results

### Neutrophilic infiltration in Behçet disease synovitis

**Figure 1:**
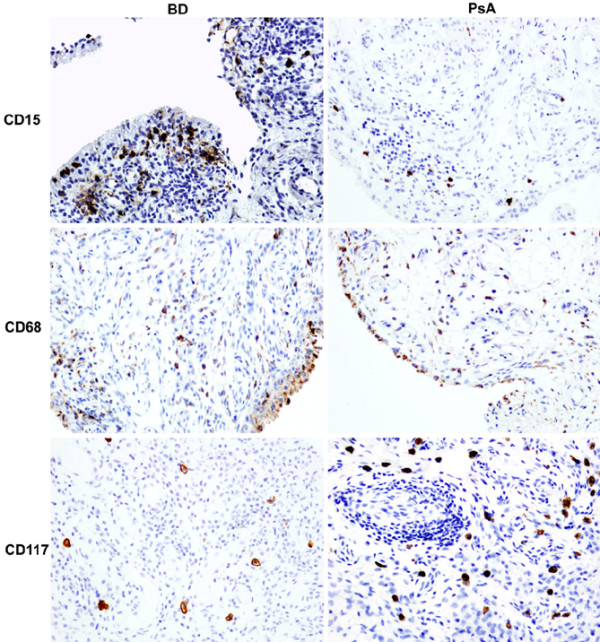
Microscopic analysis of synovial inflammation with innate immune cells in early untreated Behçet synovitis (BD) versus psoriatic synovitis (PsA). Whereas the global degree of synovial inflammation, reflected by the number of CD68^+ ^macrophages, was similar in the two diseases, there were significant increases of CD15^+ ^neutrophils in BD and of CD117^+ ^mast cells in PsA. Original magnification ×20.

The results of the immunohistochemical analysis of the synovial tissue samples are summarized in Table 1. The number of CD68^+ ^macrophages, which reflects global synovial inflammation [16], was similar in the intimal lining layer as well as the synovial sublining of BD and PsA, indicating that there is no systematic bias in local inflammation between the two study groups (Figure 1). Further analysis of the synovial leukocyte infiltration revealed two striking differences between BD and PsA in non-lymphocytic innate immune populations. First, there was an important infiltration with CD15^+ ^neutrophils in BD, representing approximately 13% of the total synovial cellularity compared with approximately 4% in PsA (Figure 1). This neutrophilic infiltration in BD synovitis was seen essentially in the intimal lining layer (*P *= 0.036 versus PsA), with differences that were less pronounced in the synovial sublining. This prominent neutrophilic infiltration was not related to higher levels of IL-8, a major chemoattractant for neutrophils, in BD versus PsA (Table 2). Second, this increase in neutrophils was a specific rather than a general phenomenon related to innate immune cells as the number of cells expressing CD117 (c-kit), which is abundantly expressed on mast cells, was decreased in BD versus PsA (*P *= 0.046). Finally, there was no difference in the number of CD31^+ ^blood vessels between BD and PsA. There were no signs of neutrophilic or cytoclastic vasculitis.

**Table 1:** Immunohistochemical analysis of synovial tissue biopsies from early untreated Behçet disease and psoriatic arthritis

	Behçet disease		Psoriatic arthritis		*P *value
			
	n = 8		n = 9		
Synovial immunopathology (cells/mm^2^)					

CD68 lining	3,681	(877–6,831)	2,947	(541–4,834)	NS
CD68 sublining	529	(311–2,339)	739	(183–2,636)	NS
CD3	1,077	(354–1,427)	336	(164–1,036)	0.015
CD4	516	(31–1,306)	196	(3–1,061)	NS
CD8	524	(2–832)	113	(3–669)	NS
CD20	126.5	(61–703)	106	(38–510)	NS
Lymphoid aggregates	0.93	(0.34–6.8)	0.43	(0–3.04)	NS
Grade-3 lymphoid aggregates	0.08	(0–1.38)	0	(0–0.44)	NS
CD138	45	(11–432)	173	(8–1,490)	0.071
CD56	17	(1–144)	9	(2–77)	NS
Granzyme B	12	(2–93)	9	(2–20)	NS
Perforin	8.5	(3–26)	6	(1–22)	NS
CD15 lining	919	(101–4,436)	275	(11–1,015)	0.036
CD15 sublining	305.5	(31–1,050)	203	(25–467)	NS
CD117	60	(21–286)	162	(29–365)	0.046
CD31 (vessels/mm^2^)	80	(60–255)	133	(63–166)	NS

After immunostaining of paraffin or frozen sections, the number of positive cells per square millimeter was assessed by digital image analysis. Data are presented as median (range). NS, non-significant.

**Table 2:** Synovial fluid analysis in early untreated Behçet disease and psoriatic arthritis

	Behçet disease		Psoriatic arthritis		*P *value
			
	n = 8		n = 9		
Cells/mm^3^	15,840	(7,600–30,720)	15,000	(2,250–35,500)	NS
Neutrophils/mm^3^	10,865	(4,200–28,000)	10,000	(562–23,450)	NS
Percentage neutrophils	72.5	(53.75–91.15)	74.07	(24.98–94)	NS
IL-6	8,850	(6,484–12,157)	8,678	(6,107–11,022)	NS
IL-8	272	(115–4,500)	241	(102–3,033)	NS
TNF-α	13.5	(9–44)	11	(0.1–32)	NS
IFN-γ	103.5	(9–323)	89	(62–134)	NS
IL-2	26.5	(9–34)	38	(31–78)	0.017
IL-4	80.5	(66–111)	86	(77–107)	NS
IL-10	17.5	(5–22)	10	(2–28)	NS
IL-17	<25		<25		NS
Perforin	1168	(428–2,010)	278	(132–642)	0.005
Granzyme B	<15	(<15–56)	<15	(<15–41)	NS

The cytokines as well as perforin and granzyme B were detected by enzyme-linked immunosorbent assay and are expressed as picograms per milliliter. Data are presented as median (range). IFN-γ, interferon-gamma; IL, interleukin; NS, non-significant; TNF-α, tumour necrosis factor-alpha.

### Accumulation of T lymphocytes in Behçet disease synovitis

**Figure 2:**
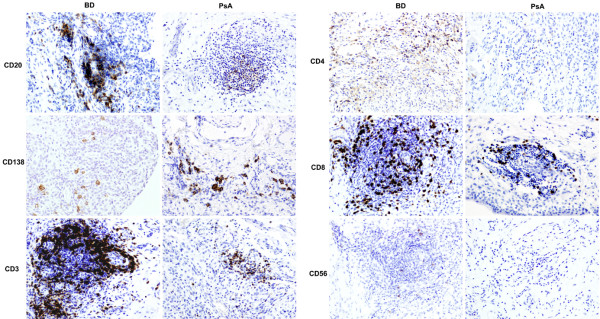
Microscopic analysis of synovial inflammation with lymphocytes in early untreated Behçet synovitis (BD) versus psoriatic synovitis (PsA). Representative pictures are shown of the immunostainings for CD20^+ ^B lymphocytes; CD138^+ ^plasma cells; CD3^+^, CD4^+^, and CD8^+ ^T lymphocytes; and CD56^+ ^natural killer cells. The number of infiltrating CD3^+ ^T lymphocytes was significantly increased in BD versus PsA. Original magnification ×20.

In the analysis of the lymphocytic infiltration (Table 1), the numbers of CD20^+ ^B lymphocytes, lymphoid aggregates, and large grade-3 aggregates were similar in the two diseases, with a trend toward lower CD138^+ ^plasma cell numbers in BD versus PsA (*P *= 0.071) (Figure 2). In sharp contrast, there was a threefold increase of CD3^+ ^T lymphocytes in BD versus PsA (*P *= 0.015) (Figure 2). Though not statistically significant, this increase was seen in both the CD4^+ ^and CD8^+ ^T-lymphocyte subsets.

### Synovial fluid cytokine profiles

As the immunohistochemical analysis indicated a striking difference in the number of infiltrating T cells between BD and PsA synovitis, we next aimed to analyze the Th1/Th2/Th17 profile by assessing SF cytokines. As shown in Table 2, BD and PsA had similar SF leukocyte and neutrophil counts, reflecting a similar degree of local inflammatory reaction in the two diseases. This was further confirmed by the similar levels of IL-6. In an investigation of skewing toward Th1 (IFN-γ, TNF-α, and IL-2), Th2 (IL-4 and IL-10), or Th17 (IL-17) cytokine profiles, only IL-2 showed significantly higher levels in PsA than in BD (*P *= 0.017) (Table 2).

### Increased synovial fluid perforin levels in Behçet disease

As we observed no clear skewing of the Th1/Th2 profile, we next investigated whether the infiltrating T cells displayed features of cytotoxic cells. CD56, a marker expressed by cytotoxic T cells and natural killer (NK) cells, was slightly but not significantly increased in BD versus PsA (Figure 2). Immunostaining of perforin and granzyme B, both cytotoxic effectors, showed only few positive cells without differences between BD and PsA (Table 1), but the SF levels of perforin showed a highly significant four- to fivefold increase in BD (1,168 pg/mL, range 428 to 2,010 pg/mL) versus PsA (278 pg/mL, range 132 to 642 pg/mL) (*P *= 0.005) (Table 2). The SF levels of granzyme were below the detection limit of the ELISA in all samples.

## Discussion

Although BD is a relatively common and potentially severe systemic disease, the pathophysiology of the disease remains poorly understood. Microbial infections, sterile neutrophil-mediated inflammation, and autoimmune lymphocytes have all been implicated in the recurrent attacks of inflammation characteristic of this disease [1], leading to an ongoing debate on the autoimmune versus autoinflammatory origin of BD [17]. As we and others demonstrated previously that detailed analysis of the histopathology of the target lesions can yield important pathophysiological information in rheumatic conditions [2-4,10], we took advantage here of the predilection of the disease for knee joints and the availability of biopsy sampling by needle arthroscopy to revisit in detail the synovial immunopathology of BD. None of the arthroscopies, performed primarily for diagnostic and/or therapeutical reasons, leads to cutaneous or joint complications, including pathergy-like reactions [18]. This observation indicates that it is medically and ethically acceptable to perform histology studies in BD.

Almost 30 years ago, a study a study assessed for the first time the histology of synovitis in six BD patients [5]. That study found no evidence of infection but a dense mixed inflammatory infiltrate with the prominent presence of neutrophils and scarcity of plasma cells in most samples. Similarly, a second study found a paucity of plasma cells in 12 BD synovial tissues but did not describe the presence of neutrophils [7]. Strikingly, they found ectopic lymphoid neogenesis, defined as the development of large lymphoid aggregates resembling secondary lymphoid organs [12-15], in 5 out of 12 samples. Unfortunately, the interpretation of both data sets is hampered by the inclusion of patients with long disease duration and the lack of a control group with non-BD synovitis. Including RA synovial tissue as control, a third study could not confirm the prominent infiltration with neutrophils or any other distinctive feature in seven BD cases but indicated that the findings may be influenced by the longer disease duration [6]. The present study was designed to overcome the limitations of these previous analyses by systematic comparison with a clinically related inflammatory arthritis, PsA, and by stringent selection of active, early, and untreated disease for both groups. As local disease activity is an important determinant of synovial histopathology [10], this design additionally allowed us to match BD and PsA for local disease activity as measured by SF cell count, SF IL-6 levels, and synovial tissue CD68^+ ^macrophage numbers [16]. As such, this stringent study design obviously limits the number of cases that can be included but minimizes the risk of systematic biases and false-positive findings.

In the analysis of the infiltrating leukocytes from the innate immune system, a first striking feature of BD synovitis was the marked infiltration with polymorphonuclear neutrophil (PMN). This neutrophilic infiltration cannot be explained by disease duration as both BD and PsA had very early, untreated disease. It was also not related to a difference in levels of IL-8, a major chemokine for PMN. Moreover, this increase did not extend more broadly to innate immunity in general as cells positive for the mast cell marker CD117 appeared to be decreased in BD versus PsA. This relative increase in BD versus PsA most likely reflects a true increase in BD as previous studies showed that the number of infiltrating PMN was already high in PsA compared with RA synovial tissue [3,4]. Increased numbers of PMN in BD inflammation have been reported not only in synovium [5] but also in other target organs such as skin [19,20] and the central nervous system [21]. Moreover, the function of PMN has been reported to be altered in BD [22,23]. Taken together, these data are consistent with a prominent role of PMN in the disease process and may suggest some degree of similarity between the pathophysiology of the synovitis and skin reactions such as the pathergy test in BD. In contrast, however, we did not find any evidence for neutrophilic vasculitis or any other form of vessel pathology in the synovial tissue. Synovitis is thus clearly different from cutaneous lesions in this respect as leukocytoclastic vasculitis is a prominent feature of skin lesions in BD [24,25].

A second important finding was the selective increase of synovial T lymphocytes in BD. Again, this was specific for T cells rather than related to a global increase in lymphocyte infiltration as we found similar numbers of B lymphocytes and a similar degree of organization in lymphoid aggregates in both diseases. Moreover, previous studies showed no difference in T-cell infiltration between PsA and RA, suggesting that the increase in BD is actually specific [3,4]. Confirming previous studies on BD synovitis [5,7], plasma cells were even lower in BD versus PsA synovitis. Although we did not investigate the mechanism underlying the selective increase in T lymphocytes, previous studies indicated that BD T cells are partially protected against apoptosis [26]. Moreover, a recent report indicates that specific T-cell subsets are associated with sterile neutrophil-rich inflammation as observed in BD synovitis, which may explain the simultaneous increase of both T cells and PMN [27].

As to the potential functional contribution of these T cells to the pathology, both Th1 skewing [28,29] and cytotoxicity by classical CD8^+ ^cells, NK T cells, or gamma-delta cells [28-30] have been proposed. Our SF analysis failed to demonstrate a clear Th1 skewing, with lower IL-2, but not for IFN-γ and TNF-α, levels in BD versus PsA. There was also no significant difference in the number of CD8^+^, CD56^+^, perforin^+^, or granzyme B^+ ^cells, defining cytotoxic cells, in BD versus PsA synovitis. However, the levels of perforin released in the SF were fivefold increased in BD versus PsA, suggesting the relevance of cytotoxicity in BD synovitis [30-32].

## Conclusion

This systematic comparative analysis of early untreated synovitis identifies both lymphocytes and PMN as important infiltrating cell populations and indicates an increase of cytotoxic molecules in BD versus PsA. Further studies should clarify their functional contribution to the pathogenesis of the disease.

## Abbreviations

BD: Behçet disease; ELISA: enzyme-linked immunosorbent assay; IFN-γ: interferon-gamma; IL: interleukin; NK: natural killer; PMN: polymorphonuclear neutrophil; PsA: psoriatic arthritis; RA: rheumatoid arthritis; SF: synovial fluid; SpA: spondyloarthritis; TNF: tumor necrosis factor.

## Competing interests

The authors declare that they have no competing interests.

## Authors' contributions

JDC helped to design the study, to collect the samples, to perform the experiments, to analyze the data, and to prepare the manuscript. DB helped to design the study, to analyze the data, and to prepare the manuscript. RC, TN, CM, JAG-P, PP, and AP helped to collect the samples and to perform the experiments. PPT and RS helped to prepare the manuscript. All authors read and approved the final manuscript.
